# TRANSITION OF HEALTH PROFILES IN COGNITIVELY INTACT NURSING HOME RESIDENTS WITH SUICIDAL IDEATION

**DOI:** 10.1093/geroni/igac059.2852

**Published:** 2022-12-20

**Authors:** Yiyang Yuan, Adrita Barooah, Deborah Mack, Kate Lapane, Anthony Rothschild, Christine Ulbricht

**Affiliations:** University of Massachusetts Chan Medical School, Worcester, Massachusetts, United States; University of Massachusetts Boston, Boston, Massachusetts, United States; University of Massachusetts Chan Medical School, Worcester, Massachusetts, United States; University of Massachusetts Chan Medical School, Worcester, Massachusetts, United States; UMass Chan Medical School, Worcester, Massachusetts, United States; Takeda, Cambridge, Massachusetts, United States

## Abstract

For older adults entering nursing homes with self-reported suicidal ideation (SI), little is known about how their health profiles change during the first 90 days post admission. Using the 9th Patient Health Questionnaire-9 item on Minimum Data Set 3.0, we identified 5,397 cognitively intact, older adults with SI at admission, 29% of whom continued to report SI at 90 days. Latent transition analysis identified five health profiles at admission: (1) frail, severe pain, all depressive symptoms (prevalence: 25%); (2) frail, moderate pain, depressed mood, insomnia/hypersomnia, fatigue (27%); (3) frail, no pain, depressed mood, fatigue, and worthlessness (18%); (4) pre-frail, no pain, depressed mood (15%); (5) robust, no pain, depressed mood, insomnia/hypersomnia, fatigue (15%). At 90 days, while the profiles remained mostly the same, improvement in depressive symptoms and pain were observed: (1) frail, severe pain, most depressive symptoms (17%); (2) frail, no pain, depressed mood, and fatigue (28%); (3) frail, no pain, depressed mood, fatigue, and worthlessness (15%); (4) pre-frail, no pain, no depressive symptoms (25%); (5) robust, no pain, depressed mood, and fatigue (16%). Most residents remained in the same profile over time. Of those who were in Profile (1) at admission, 20% transitioned into Profile (2), 12% to Profile (4), and 7% to Profile (5). This work highlights the need to now investigate the potential drivers for specific profile transitions among nursing home residents presenting with SI on admission, thereby informing interventions for improved identification and treatment of SI in the nursing home setting.

